# Autograft or allograft for reconstruction of anterior cruciate ligament: a health economics perspective

**DOI:** 10.1007/s00167-019-05436-z

**Published:** 2019-03-14

**Authors:** Hema Mistry, Andrew Metcalfe, Jill Colquitt, Emma Loveman, Nick A. Smith, Pamela Royle, Norman Waugh

**Affiliations:** 1 Division of Health Sciences, Warwick Medical School, Gibbet Hill Campus, University of Warwick, Coventry, CV4 7AL UK; 2 Warwick Clinical Trials Unit, University of Warwick Medical School, Coventry, CV4 7AL UK; 3 Effective Evidence, Waterlooville, Hampshire, PO8 9SE UK; 4 Department of Orthopaedics, University Hospitals Coventry and Warwickshire, Coventry, CV2 2DX UK

## Abstract

**Purpose:**

To assess the clinical and cost-effectiveness of allografts versus autografts in the reconstruction of anterior cruciate ligaments.

**Methods:**

Systematic review of comparative clinical effectiveness and cost-effectiveness analysis.

**Results:**

Both autograft and allograft reconstruction are highly effective. Recent studies show little difference in failure rates between autografts and allografts (about 6% and 7%, respectively). In cost-effectiveness analysis, the price differential is the main factor, making autografts the first choice. However, there will be situations, particularly in revision ACL reconstruction, where an allograft may be preferred, or may be the only reasonable option available.

**Conclusion:**

In ACL reconstruction, clinical results with autografts are as good as or slightly better than with allografts. Allografts cost more, indicating that autografts are more cost-effective and should usually be first choice.

**Level of evidence:**

II.

## Introduction

Rupture of the anterior cruciate ligament (ACL) is not uncommon in people taking part in sports. A full description of the clinical aspects is provided in another article in this issue, by Hulet et al. [20]. ACL rupture can sometimes be managed conservatively but results are considered better with reconstruction, especially regarding stability, although further trials are ongoing. Non-operative approaches may be considered in lower demand patients but may result in ongoing instability, and are not cost-effective in sportspeople [38], whereas ACL reconstruction gives good results, and allows people to get back to vigorous, and indeed international level, sport. A cost-effectiveness analysis by Stewart et al. [40] concluded that ACL reconstruction was cost-effective compared to physiotherapy and no reconstruction in competitive athletes.

Some people do try to repair the ACL, but results do not so far seem to have been good. This article is only concerned with reconstruction.

Autografts can come from different source tendons. The commonest source now seems to be hamstring tendons but some surgeons prefer bone–patellar tendon–bone (BPTB) as first line, and others use BPTB in high-risk patients.

Allografts come from various sites, including tibialis anterior, quadriceps, Achilles tendon, BPTB and hamstrings (HS).

As noted by Hulet et al. [20], practice varies, with allografts used much more often in some countries than others. Prentice et al. [37] reported practice in six registries, in Denmark, Luxembourg, Norway, the UK, Sweden and the USA, with data from 101,125 procedures (95% from Scandinavian countries, 4% from the UK). In Europe, allografts were used in few patients (0.3–6.3%), whereas they were used in 40% in the US centres (Kaiser Permanente). Revision rates by 7 years were reported for Norway (5.6%), Sweden (4.1%) and KP (6.1%).

The advantages of allografts are no donor site morbidity, a shorter operation and less painful initial recovery. The disadvantages are slower graft incorporation and concern about higher rupture rates in some young highly active groups, concern about disease transmission and increased cost. See review by Hulet et al. [20]. The concern about a higher re-rupture rate may not be warranted and may date from the time when allografts were irradiated or chemically cleansed, and weakened by that. Disease transmission is now rare following more rigorous testing of donors.

## Materials and methods

Recent good-quality systematic reviews were identified first, followed by any recent trials not included in those reviews. A high-quality, recent review of both systematic reviews and randomised trials (RCTs) by Zeng et al. [52] was identified comparing allografts and autografts in primary ACL reconstruction. This review was used as the starting point and only primary studies that were published since the dates of its searches in 2014 were included. For completeness, eight other good-quality recent systematic reviews were examined. Wasserstein et al. [45], Kan et al. [21] and Cvetanovich et al. [12] were reviews of primary ACLs. A review by Mascarenhas et al. [29] was a review of meta-analyses. Yao et al. [50] included only studies before 2014 so was not discussed further. Bansal et al. [2] was a good-quality review but specifically on infections. It reported a much higher rate with HS autografts than with BPTB autografts, though this was based on observational studies, but no difference overall between autografts and allografts. Wei et al. [47] compared autografts with non-irradiated allografts. Park et al. [36] also focused on the irradiation issue. This article does not discuss radiation further as it is covered in the review by Hulet et al. [20].

Two reviews by Grassi et al. [17] and Mohan et al. [30] considered revision ACL reconstruction. Grassi et al [17] found that autografts gave better results than allografts in revision ACL reconstruction. Whereas, Mohan et al. [30] found revision ACL reconstruction had failure rates similar to autograft or allograft reconstruction.

*Cost-effectiveness analysis* A decision tree model in Microsoft Excel® was considered the most appropriate choice as ACL reconstruction is usually successful and most patients return to a functioning knee after reconstructive surgery. The starting point for the economic model is the decision to do an ACL reconstruction; the model does not include a non-reconstruction arm. The clinical pathways were developed using information from the published literature and clinical experience. Further information on the economics model including the structure is provided in the full report on the ESSKA website.

### Base-case analysis

For the base-case analysis, a 3-year time horizon was adopted. The analysis does not differentiate by gender nor mortality is taken into account. The starting age for a patient is 25 years. The analysis is conducted from the perspective of the UK National Health Service (NHS) and personal social services (PSS). All costs are in pounds sterling (£) in 2016/2017 prices. Health outcomes are measured in quality-adjusted life years (QALYs). Results are expressed as incremental cost-effectiveness ratio (ICER) more commonly known as a cost per QALY gained. An annual discount rate of 3.5% is applied to both costs and outcomes in line with recommended guidelines [31].

#### Resource use and costs

All unit costs reported in Table 1 are presented in pounds sterling (£) in 2016/17 prices. The cost of the allograft (£2250) was based on the NHS Tissue Services price list for 2018/19 [32] and was an average of the prices of the tendons likely to be used, such as the tibialis anterior one (which once doubled is the same size or thicker than the combined semitendinosus and gracilis graft). The exact prices are classed as confidential. There is no cost for the graft in the HS autograft arm.

**Table 1:** Resource use and costs for ACL reconstruction

Resource use	Unit cost (£)	Source
*Graft type*		
Allograft	£2250	[32]
*Procedure*		
Intermediate knee procedures for non-trauma, 19 years and over (HRG code: HN24C)	£1642	[15]
*Other related costs*		
Three consultant led outpatient follow-up attendance (HRG code: WF01A)	£336	[15]
8 hospital physiotherapy sessions (30 min)	£132	[10]
Paracetamol (two tablets twice a day per year)	£23.21	[5]
Ibuprofen (one tablet a day per year)	£12.47	[5]
***Total costs***		
Allograft	£4395	
HS autograft	£2145	
BPTB autograft	£2145	
*Infection*		
Infections	£7761	[13]
*Conservative care*		
One consultant led outpatient follow-up attendance (HRG code: WF01A)	£112	[15]
8 hospital physiotherapy sessions (30 min)	£132	[11]

It was assumed that 0.3% of all reconstructions will get infections based on a recent ACL study by Waterman et al. [46]. The cost of infections was obtained from the Genuario et al. paper [13] in US $ in 2010 prices. Costs into UK £ in 2017 prices were converted using the World Bank gross domestic product (GDP) deflators [48] and the purchasing power parity (PPP) measures [34]. The cost of treatment for an infection included the cost of debridement, irrigation, and antibiotics started intravenously with 1-week hospital admission then continued for a further 5 weeks [3].

**Fig. 1:**
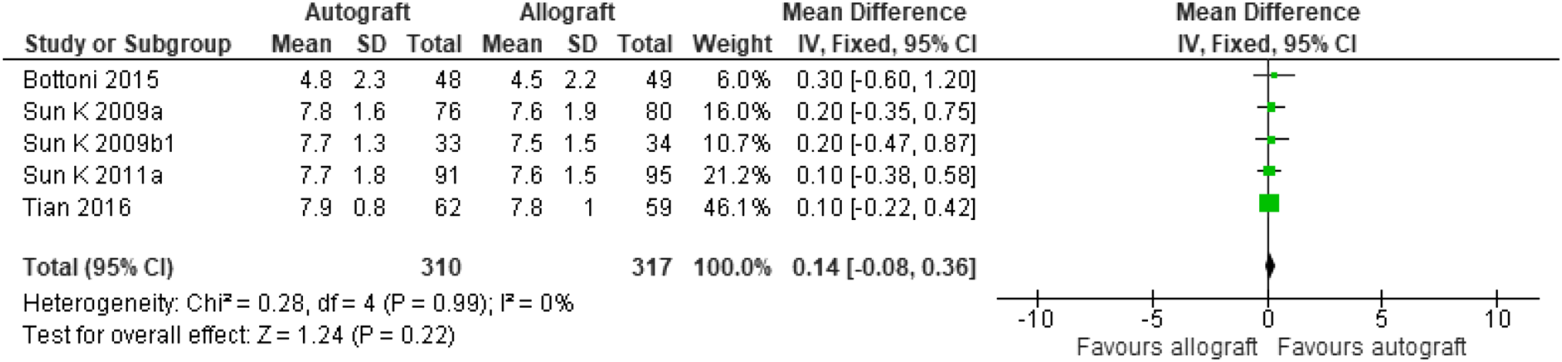
Updated meta-analysis of non-irradiated allografts versus autografts, Tegner score

For the base-case analysis, probabilities for the decision model were obtained from the literature and clinical expert opinion (see Table 2). For the 1st ACL reconstruction, these probabilities were obtained from a systematic review and meta-analysis of randomised controlled trials which focused on autograft versus allograft in ACL reconstruction by Zeng et al. [52]. Five trials were included in the forest plot meta-analysis for the subgroup analysis of autograft versus non-irradiated allograft failure (Fig. 1) ([24, 33, 41–43]). There were 16 events in the autograft arm which resulted in a failure rate of 5.57% (*n* = 287) and there were 20 events in the allograft arm with a failure rate of 6.92% (*n* = 289).

**Table 2:** Probabilities for ACL reconstruction

Pathway	Probability	Source
*Allograft pathway*		
1st Allograft		
Success	0.931	[50, 52]
Fail	0.069	
2nd or 3rd Allograft		
Success	0.964	[30]
Fail	0.036	
*HS autograft pathway*		
1st HS Autograft		
Success	0.944	[52]
Fail	0.056	
2nd or 3rd Allograft		
Success	0.964	[30]
Fail	0.036	
2nd or 3rd BPTB Autograft		
Success	0.959	[30]
Fail	0.041	
2nd or 3rd HS Autograft (other leg)		
Success	0.959	[30]
Fail	0.041	

For the second ACL reconstruction, probabilities were obtained from the paper by Mohan et al. [30], who conducted a random effects meta-analysis of clinical outcomes in revision ACL reconstruction. The primary outcome was graft failure. Eight studies with a combined number of 2302 patients provided an autograft failure rate of 4.1% (95% CI 2.0–6.9%) and two studies with a combined number of 671 patients provided an allograft failure rate of 3.57% (95% CI 1.38–6.74%).

### Utilities

For patients who have a successful ACL reconstruction, population norm values provided by Ara and Brazier [1] were used. Genuario et al. [13] reported utility values for different types of graft for ACL reconstruction. For patients in whom either an autograft or an allograft fails or who are in the conservative care arm, a utility value of 0.790 was assigned, which corresponded to the instability health state. For the few patients who get an infection, a disutility value for 6 weeks was applied. These utility values were then weighted by the length of time in that health state to estimate QALYs.

## Results

Zeng et al. [52] reviewed systematic reviews and RCTs comparing allografts and autografts in people having primary ACL reconstruction. The review of reviews included ten systematic reviews and nine RCTs. The review was assessed as very high quality (eight of eight quality items rated as ‘yes’). Autograft versus allograft (some of which used irradiated grafts) had a pooled risk ratio on the overall International Knee Documentation Committee (IKDC) level ‘normal and nearly normal’ of 1.03 (95% CI 1.00, 1.07); *p* = 0.03, in favour of autograft. There was no statistical heterogeneity (*I*^2^ 0%). However, when two studies were excluded in sensitivity analyses (the review examined single-study influence on results by removing one at a time) the pooled RR was no longer statistically significant. Clinical failure was also less frequent (RR, 0.47; 95% CI 0.31, 0.73; *p* = 0.0007; *I*^2^ 23%), and Tegner scores (WMD, 0.36; 95% CI 0.11, 0.60; *p* = 0.004; *I*^2^ 0%) differences were also statistically significant, but the Lysholm score was not (weighted mean difference, WMD, 0.02; 95% CI − 0.71, 0.75; non-significant (n.s.); *I*^2^ 44%). These analyses included studies using irradiated allografts. Subgroup analyses of autograft versus non-irradiated allografts were also reported for these outcomes, none of which were statistically significant (Lysholm WMD, − 0.64; 95% CI − 1.45, 0.17; n.s.; *I*^2^ 0%; Tegner WMD 0.16; 95% CI − 0.16, 0.47; n.s.; *I*^2^ 0%). The authors concluded that autograft had advantages over irradiated allograft with respect to function and stability, whereas there were no significant differences between autograft and non-irradiated allografts.

Mariscalco et al [28] included nine studies comparing autografts with non-irradiated allografts, four of which were RCTs or mainly RCT (one trial had 25% patient choice and 75% randomised), with the other studies mainly patient choice. They found no difference in outcomes.

One review by Lording et al [25] did report a higher failure rate with non-irradiated allografts than with autografts, but the differences from previous reviews arose mainly from the inclusion of two studies, Bottoni et al. [4], and a study in people aged under 25 [22].

One of the primary studies, Gorschewski et al. [14] reported an unusually high failure rate with BPTB allografts, with failures in 21% at 2 years and 45% by 6 years in the allograft group, compared to 5% and 6% in the autograft group. The allografts were treated with the Tutoplast methods and were irradiated.

### Failure and revisions, allografts versus autografts

Three of the primary studies reported failures and revision rates for allografts and autograft ACL reconstructions. In the RCT by Yoo et al. [51] at 33–35 months follow-up, the rates of revision were similar between groups (allografts 1.6%; autografts 1.5%). The rate of failure requiring revision was statistically significantly higher in the allograft group of the Bottoni et al. [4] RCT than the autograft group (26.5% and 8.3%, respectively, *p* = 0.03, duration of follow-up 10.5 years). In the third study by Yang et al. [49], the failure rates at 2.5 years were 2.4% with allografts and 2.2% autograft. All three studies used fresh-frozen non-irradiated allografts.

The reasons for the higher failure rate in Bottoni et al. [4] are not clear. The operations were done a long time ago, perhaps at a time when processing methods were more damaging. Grafts came from a single tissue bank over a relatively short period of time.

Concerns has been expressed by some reviewers of a higher failure rate in allografts used in patients under the age of 25 [6]. Kane et al [22] found a higher revision rate in non-irradiated allograft BPTB grafts, but the potential for selection bias in that study was very high. In the large Kaiser Permanente database [26, 44], an increased rate of failure was noted for BPTB allografts compared to autografts which may explain the perceived differences. There was no statistical difference seen when autografts were compared to non-processed soft tissue allografts in the Kaiser Permanente series, although follow-up remains relatively short for this analysis [16].

### Clinical scores

**Fig. 2:**
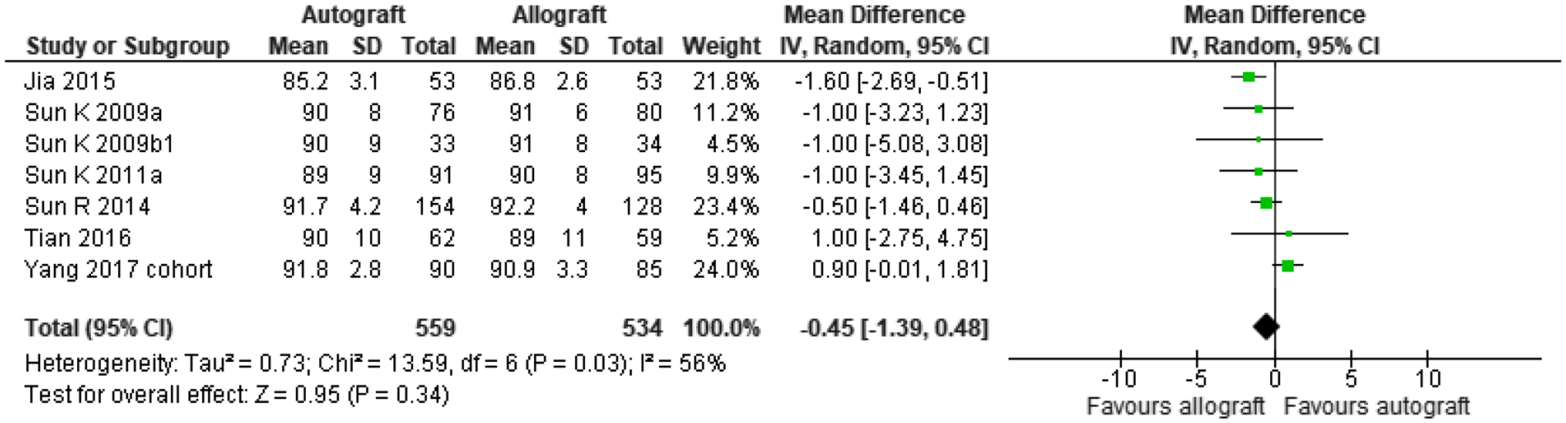
Updated meta-analysis of non-irradiated allografts versus autografts, Lysholm score

The Zeng meta-analysis [52] has been updated with the addition of some more recent studies as shown in Figs. 1 and 2.

### Adverse events

Hardy et al [19] provide a systematic review specifically on the adverse events after harvesting autografts for ACL reconstruction. They note that in France most ACL reconstructions are done with autografts, taken from hamstring tendons, patellar tendon and fascia lata. They included 36 articles in a good-quality review. For hamstring autografts, they conclude that there are complications in 8.3% of cases (though some studies have much higher rates). The commonest is saphenous nerve damage, though they think this is largely avoidable by a different approach. Temporary strength deficits (up to 3 months) occur. Because these complications are temporary, they will have insignificant impact on the long-term economics.

They estimate fewer complications with patellar tendon (PT) grafts (0.2–1.2% overall) but some more serious, including patellar fracture in 0.42–1.3%, rupture of PT and anterior knee pain, reported in as many as 46%, but with varying definitions.

To guide practice, an evidence review from New Zealand from Accident Compensation Corporation Research [39] has been produced. It was based on an overview of 12 systematic reviews. The primary studies were not examined. The last search was done in May 2016, and the reviews were published from 2007 to 2015. The ACC report concluded that there was no evidence of any significant differences in failure rates or other outcomes, between autografts and non-irradiated allografts. It concluded that allografts irradiated with low doses still performed less well than non-irradiated allografts, and that low doses were not sufficient to eliminate the risk of disease transmission. Given the similar outcomes, cost became the determining factor. It appears that costs of allografts are higher in NZ than elsewhere because there is no local provider.

Older studies may not reflect modern processing methods. Fresh-frozen allografts give better results. Mardani-Kivi et al. [27] found no difference in outcomes between fresh-frozen tibialis posterior allografts and hamstring autografts after 55 months. Krych et al. [23] reported a meta-analysis showing that BPTB autografts did better than allografts, but the advantage only applied when allografts were irradiated or chemically processed.

### Cost-effectiveness

#### Previous studies

Salzmann et al. [38] provide a review of 24 economic studies in ACL reconstruction (ACLR). They note that 17 were reports only of costs, of which five compared autograft and allograft ACLR. The other seven include three cost-utility studies of ACLR versus non-operative management, with all three concluding that surgery was more cost effective. Two studies compared the cost-effectiveness of single versus double-bundle techniques. One study compared prompt versus delayed ACLR. The remaining study by Genuario et al. [13] was the one most relevant to this review, because it compared autografts with allografts. They found that hamstring autograft was least costly and more effective, than both BPTB and allograft reconstructions.

Some studies report small differences in in-patient stays. Gorschewski et al. [14] reported mean stays of 5.2 days for allografts and 6.3 days for autografts. They also reported a slightly earlier return to work after allografts (2.3 months versus 2.6 months, *p* = 0.004).

Cooper and Kaeding [9] report hospital costs for ACL reconstruction of $4,072 for autografts and $5,195 for allografts. The slightly shorter theatre time for allografts had little effect on the cost differential.

Oro et al. [35] also report that operating time was 12 min longer with autograft but the total cost was about $1000 more with allograft.

Cohen et al. [7] report that saphenous nerve damage is commoner after ACLR with autografts, but not enough to be economically significant.

Greis et al. [18] compare costs of tibialis allografts with hamstring autografts in Utah. The mean cost of ACLR allografting was $4587 with theatre time of 92 min. The autograft cost was $3489 with 125 min of theatre time.

Cole et al. [8] report hospital charges of $4622 for allografts and $5694 for autografts. The difference is due to longer operating theatre time and longer inpatient stays for autograft patients.

One issue to be considered in interpretation of all cost-effectiveness studies is how old the clinical effectiveness data that support them are. For example, older studies may have a mixture of allografts sterilised by different methods, including radiation. The high-quality review by Zeng et al. [52] showed no difference in success rates when allografts were compared with non-irradiated grafts, but that autografts were more successful than irradiated grafts.

### Modelling

Table 3 shows the base-case deterministic results.

**Table 3:** Base-case deterministic discounted results, ACL reconstruction

Procedure	Total mean costs £	Total mean QALYs	Incremental costs	Incremental QALYs	ICER
HS autograft	£2420	2.6980	–	–	–
Allograft	£4846	2.6953	£2426	− 0.0026	Dominated

*ICER* incremental cost-effectiveness ratio

Having an allograft as a primary ACL reconstruction is more costly (£2,426 more) and no more effective (very slightly less effective at 0.0026 QALYs—though this is not clinically significant) than having a HS autograft as a primary ACL reconstruction: that is, HS autografts “dominated” allografts. The main cost driver for this result was the cost of the graft. The second but less important factor was that the failure rate for allograft was slightly higher, by 1.3% (6.9% versus 5.6%) than the HS autograft. However, this has little impact compared to allograft cost.

There are some small advantages for allografts—slightly shorter theatre time, no donor site morbidity—but these factors are too small to significantly affect cost-effectiveness.

## Discussion

When there are existing good-quality reviews, it is unnecessary and indeed wasteful to repeat them. They should simply be updated with any new trials, as has been done in this article. There have been numerous reviews and RCTs in this area, mostly with consistent results. Where there are differences in the failure rates of ACL reconstructions between allografts and autografts, these can mostly be explained by the use of irradiated grafts. Giving sufficient radiation to achieve sterility will likely weaken grafts and make them more likely to fail and, therefore, the practice of irradiating grafts is not recommended.

The evidence shows no significant differences in clinical effectiveness between autografts and non-irradiated allografts. Failure rates with both grafts are now low. BPTB allografts have been described as having higher failure rates in some studies, compared to autograft, but this does not seem to be the case for soft tissue allografts, with only one RCT showing a difference between soft tissue allograft and autografts [4], whilst the majority of RCTs had failure rates that were very similar. The RCT by Bottoni et al [4] that showed a difference had an unusually high allograft failure rate of 27%. The reasons for this are not clear. The operations were done a long time ago, perhaps at a time when processing methods were more damaging. Grafts came from a single tissue bank over a relatively short period of time.

Failure does not necessarily mean that there was something wrong with the procedure or the technology. It should be borne in mind that people having these procedures do so because they have damaged or ruptured their own tissues, perhaps by putting great demands on the knee structures, often during sport.

When an intervention has a higher cost than the comparator, but is no more clinically effective, or less effective, it is said to be “dominated” in cost-effectiveness analysis as is the case with allografts in this study. Costs with allografts are higher because of the cost of the graft, and the cost we used may be less than that in other countries. The findings of the economic modelling need to take into account the local costs of the graft, but this can be done simply based on the data shown here, as the cost of the graft was the dominant factor in the economic model.

There is less morbidity with allografts because they are not harvested from the live patient, but the disutility is transient and insufficient to change the overall conclusion on cost-effectiveness. Hardy et al [19] specifically conducted a review on the adverse events after harvesting autografts for ACL reconstruction. For hamstring autografts, they conclude that there are complications in 8.3% of cases, the commonest is saphenous nerve damage. Temporary strength deficits (up to 3 months) occur. Because these complications are temporary, they will have insignificant impact on the long-term economics [19].

Revision ACL reconstruction is also an effective operation, using the hamstring or BPTB graft from either the same leg or the other leg. This study was not directly designed to compare allograft and autograft usage for revision ACL reconstruction, but a review of the literature shows no difference in failure rate between allograft and autograft revision ACL reconstruction and, therefore, the cost of the allograft will continue to dominate the economic analysis.

There may be situations in which allografts should be considered. The review has assumed that a direct choice can be made between allografts and autografts, and that both are available. There may be situations where a satisfactory autograft is not available, for example in multi-ligament injury where available autografts will be used for other reconstructions. Also, in the setting of revision ACL reconstruction, there are various considerations regarding graft choice, due to tunnel size or position, previous usage of other grafts, and reconstructions in the other leg, that may mean that allografts would be preferred to autografts. The other population not covered sufficiently in the literature for us to draw conclusions is the elite sprinting athlete where autograft choices may be influenced by the effect of graft harvesting on their sport. However, in the majority of cases, it may be concluded that allograft ACL reconstruction with non-irradiated grafts is as safe, but more expensive then autograft ACL reconstruction, which is preferred as it is more cost effective.

## Conclusion

There is little difference in results of ACL reconstruction with autografts or non-irradiated allografts, with any advantage being with autografts. The cost is higher with allografts. So if autografts are available, allografts are not cost effective.
